# Quantitative Determination of Arsenic Species from Fruit Juices Using Acidic Extraction with HPLC-ICPMS

**DOI:** 10.1007/s12161-019-01636-y

**Published:** 2019

**Authors:** Kevin M. Kubachka, Sean D. Conklin, Cynthia C. Smith, Consuelo Castro

**Affiliations:** 1 Forensic Chemistry Center, US FDA, Cincinnati, OH 45237, USA; 2 Center for Food Safety and Applied Nutrition, US FDA, College Park, MD 20866, USA; 3 Kansas City Laboratory, US FDA, Lenexa, KS 66214, USA; 4 San Francisco Laboratory, US FDA, Alameda, CA 94502, USA

**Keywords:** Juice, Arsenic, Speciation, Mass balance, HPLC-ICPMS

## Abstract

Throughout the US Food and Drug Administration’s routine monitoring of various juice samples for elemental contaminants, a limited number of samples exhibited unexpected behavior related to the arsenic content. Juice samples were subjected to total arsenic determination and those containing arsenic > 10 μg kg^−1^ were subjected to arsenic speciation analysis using FDA Elemental Analysis Manual (EAM) 4.10 method (AOAC First Action Method 2016.04) to determine the concentration of iAs and other common organic arsenicals. For a subset of samples, the sum of the arsenic species was significantly less than the total arsenic value (i.e., mass balance < 65%), which is uncommon for a liquid-based matrix. Juice types that have exhibited this behavior include pomegranate, prune, and cherry juices. Causes for this issue were explored which ultimately led to an alternate sample preparation technique, extraction with 0.28 M HNO_3_ along with heat, which resulted in drastically improved mass balances approaching 100%. The method proved robust, with both accurate and precise measurements for multiple juice samples analyzed by a total of four laboratories. Two laboratories performed a level 3 multilaboratory validation. This work discusses various issues that were encountered, attempts to determine the source of the problem, the eventual solution in the form of a modified extraction procedure, and the multilaboratory validation results.

## Introduction

Arsenic is a well-known toxicant (Banerjee et al. 2011; Lee and Yu 2016) and has been classified by the International Agency for Research and Cancers as a class I carcinogen (IARC 2004). As is the case with many elements, the toxicity of arsenic is dependent upon its chemical form. In general, inorganic arsenic (iAs) species, including arsenite (As(III)) and arsenate (As(V)), are carcinogenic and are the primary forms found in drinking water (Mantha et al. 2017). Organic species are generally considered relatively less toxic (e.g., dimethylarsinic acid (DMA) and monomethyl arsenic acid (MMA)) or virtually non-toxic (e.g., arsenobetaine (AsB)).

The US Food and Drug Administration (FDA) continuously surveys and monitors toxic elements in various food commodities, including fruit juices, as part of the Total Diet Study and has been doing so since 1991 (Total Diet Study 2018). Since 2011, the FDA has placed increased emphasis on the testing of arsenic in foods (FDA 2013; Chen et al. 2014) with particular interest in arsenic speciation analysis of iAs, DMA, and MMA. In 2013, the FDA proposed an “action level” for inorganic arsenic (iAs) in ready-to-drink (RTD) apple juice of 10 μg kg^−1^ (FDA 2013). As part of routine monitoring of commodities, apple juice and other fruit juices which contain total arsenic concentrations greater than 10 μg kg^−1^ as determined using inductively coupled plasma mass spectrometry (ICP-MS) are subjected to arsenic speciation analysis using FDA Elemental Analysis Manual method §4.10 (EAM 4.10) (Conklin et al. 2013). The EAM 4.10 uses high-performance liquid chromatography (HPLC) with ICP-MS detection to determine the concentration of iAs as sum of As(III) and As(V), along with DMA and MMA. This method was recently granted AOAC First Action Status (2016.04) supported by multilaboratory validation (MLV) involving eight laboratories (Kubachka et al. 2017). The validation was performed using apple, pear, and grape (white and purple) juices. Other juice types have been analyzed, including a smaller number of cherry, cranberry, berry blend, and red grape juice during the initial EAM 4.10 development by Conklin and Chen (Conklin and Chen 2012). Additional unpublished speciation results of juices including kiwi and pomegranate have been analyzed by this method without any significant difficulties during analysis. Due to this supporting data, the scope of the EAM 4.10 is defined as clear (free of solids) fruit juices and fruit juice concentrates.

Because the EAM 4.10 is being utilized for regulatory analysis, there are many quality control measures. Mass balance is one such measure that the speciation community places great importance upon (Clough et al. 2016). This is commonly defined as the comparison of the sum of the elemental concentration of individual species determined by speciation analysis compared with the total elemental concentration of the respective element. One hundred percent mass balance is ideal; the EAM 4.10 has a control limit for mass balance of 65–135%. In our experience, juice samples for which the mass balance value is near either extreme of this range often exhibit total arsenic and/or individual arsenic species concentrations near the quantitation limits of their respective methods. According to results presented in FDA’s survey of 94 ready-to-drink (RTD) apple juice samples (Arsenic in Apple Juice), all samples with total arsenic concentrations ≥ 10 μg kg^−1^ had mass balances ≥ 75%. Additionally, samples used for the MLV and the single laboratory validation (Conklin and Chen 2012) all showed mass balances between 65 and 110%. As surveillance increased, a few samples with total arsenic concentrations greater than 10 μg kg^−1^, but with mass balances less than 65% were noted, sparking additional investigation. To date, juice types analyzed by the FDA laboratories exhibiting this trend to varying degrees include prune, pomegranate, and cherry juice (based on limited sampling).

There are very few reports in the literature that include analysis of arsenic species in prune, pomegranate, and/or cherry juice (Conklin and Chen 2012; Ellingson et al. 2014; Wang et al. 2015); although data is limited, none noted significantly deficient mass balances (< 65%). For analysis of juice samples in general, sample preparation procedures include: dilution with water and filtration (Conklin and Chen 2012; Dewi et al. 2016; Narukawa et al. 2018) with optional centrifugation (Wang et al. 2015); degassing, C18 cleanup, and syringe filtration (Coelho et al. 2005); dilution with 0.32 M HNO_3_ and filtration (Ellingson et al. 2014); and digestion with nitric acid and filtration (Roberge et al. 2009). Some of these methods reported mass balances < 65% for various juice types with no discernable pattern. The goal of this work was to develop a method that quantitatively recovers arsenic species for low mass balance juices (LMBJ). Once the optimized method was developed, a multilaboratory validation (MLV) exercise was performed.

## Materials and Methods

### Reagents and Standards

All solutions were prepared using deionized water (DIW) with resistance > 18 mΩ • cm from a Milli-Q Element system (Millipore, Bedford, MA). Ammonium phosphate (dibasic) was purchased from Sigma-Aldrich (St. Louis, MO). Ammonium hydroxide, which was used to adjust the mobile phase pH, and concentrated nitric acid were obtained from Fisher Scientific (both as Optima grade, Pittsburgh, PA). As(III) and As(V) were purchased as 1000 mg L^−1^ standard solutions from Spex Certiprep (Metuchen, NJ). Monosodium acid methane arsonate sesquihydrate (MMA) and dimethylarsinic acid (DMA) were purchased from Chem Service (West Chester, PA). Arsenobetaine (AsB) was obtained from Fluka Analytical (St. Louis, MO).

For total arsenic measurements, a certified multielement mixture standard solution including 10 μg mL^−1^ arsenic (Inorganic Ventures, Christiansburg, VA, USA) was used to prepare calibration standards. A second lot of a 100 μg mL^−1^ mixed elemental standard including arsenic (Inorganic Ventures) was used to prepare a midlevel check standard solution. An internal standard solution was prepared from internal standard solution mixture containing 10 μg mL^−1^ germanium solution (Inorganic Ventures). For speciation and total arsenic analysis, 1643f Trace Elements in Water standard reference material (SRM) was obtained from the National Institute of Standards and Technology (NIST, Gaithersburg, MD). The SRM 3035, Arsenic Species in Apple Juice, from NIST became available and was obtained after the initial validation procedures; therefore, it was analyzed in later experiments to verify method performance. The certified arsenic concentrations (± expanded uncertainty) for DMA, MMA, and iAs are 5.23 ± 0.26, 9.38 ± 0.64, and 11.53 ± 0.66 μg kg^−1^, respectively.

### Safety Information

Appropriate personal protective equipment including safety glasses, gloves, and lab coat were utilized when handling standards (powders and/or concentrated solutions) containing toxic arsenic compounds. Additionally, proper ventilation and other physical safeguards should be in place when handling said compounds. Nitric acid is highly corrosive and ammonium hydroxide is caustic; both should be handled appropriately.

### Instrumentation

Four laboratories participated in this endeavor, each with different analysis roles, but all used similar equipment as described below. The FDA, Center for Food Safety and Nutrition laboratory (CFSAN, College Park, MD) and FDA, Forensic Chemistry Center (FCC, Cincinnati, OH) performed most of the initial development work. The US FDA, Kansas Laboratory (KAN, Lenexa, KS) and San Francisco Laboratory (SAN, Alameda, CA) carried out a two-laboratory validation study of the optimized method using speciation analysis, while the former also assisted with method development. Chromatographic separations were performed on an Agilent 1260 liquid chromatographic system (Agilent Technologies, UK). Unless otherwise noted, all speciation analyses used chromatographic conditions as described in the EAM 4.10, including the use of a Hamilton PRP-X100 anion exchange column (4.1 × 250 mm, 10 μm) with matching guard column (Hamilton, Reno, NV). In summary, these conditions include a mobile of 10 mM ammonium phosphate dibasic in water with the pH adjusted to 8.25 using NH_4_OH, mobile phase flow rate of 1.0 mL min^−1^ and an injection volume of 100 μL. The column effluent was connected directly to the nebulizer of the detector.

For both arsenic total and speciation analyses, an Agilent 7700, 7900, or 8800 inductively coupled plasma-mass spectrometer (ICP-MS, Agilent Technologies, UK) was used as a mass specific detector for ^75^As. The instruments were equipped with a Scott-type double-pass quartz spray chamber, a MicroMist concentric glass nebulizer (Glass Expansion, West Melbourne, Australia) and nickel sampler, and skimmer cones. The plasma conditions for each ICP-MS instrument were optimized based on the individual laboratories’ tuning procedures before use. For all analyses, each instrument was operated using the collision−reaction cell (CRC) with helium gas mode to minimize possible polyatomic interferences, specifically ^40^Ar^35^Cl, which for speciation analysis has been previously shown (Conklin and Chen 2012) to elute fully resolved from the analytes of interest. For total arsenic analysis, the utilized He flow was between 4.0 and 5.5 mL min^−1^, and for speciation, 2.0 mL min^−1^ was used. Higher He flow rates were optimized for total arsenic analysis to yield minimum ^40^Ar^35^Cl background while preserving ^75^As signal since all sample components are simultaneously ionized with the plasma source during total ICP-MS analysis. Lower flow rates for speciation allow for higher signal intensity as well as a more stable (relative standard deviation) background which improve integration and peak detection as interferences are partially eliminated by the upfront chromatography.

For sample heating, a DigiPREP MS block digestion system (SCP Science, Quebec, Canada) was used. For filtering samples, 0.45-μm Nylon or Polytetrafluoroethylene (PTFE) syringe filters with polypropylene housing and Luer-Lock inlet were used.

### Sample Selection

**Fig. 1:**
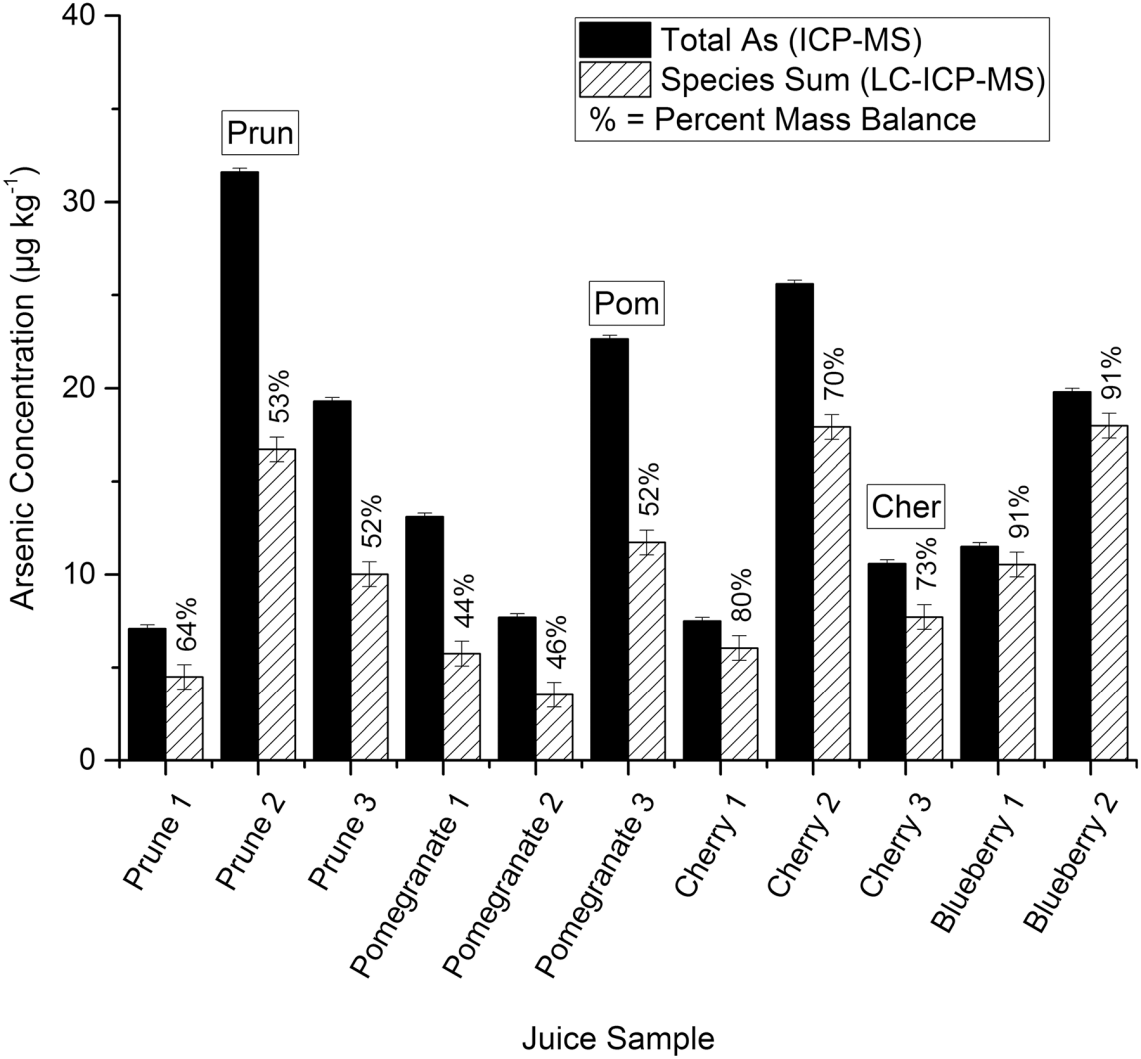
Locally purchased prune, pomegranate, cherry, and blueberry juices analyzed for total arsenic (using ICP-MS with method EAM 4.7) and arsenic species (using HPLC-ICP-MS with method EAM 4.10). For both total arsenic and speciation, the error bars represent ± one standard deviation (*σ*) based on one sample selected for triplicate analysis. Percent mass balances (species sum/total As) are noted for each sum of species bar. Samples chosen for further analysis during method development were designated Prun, Pom, and Cher

As previously mentioned, juices are routinely collected by FDA personnel and analyzed. Of the samples determined to be LMBJs, one particularly noteworthy sample was a pomegranate juice (designated Pom-Coll). Upon initial analysis, Pom-Coll exhibited one of the lowest mass balances (52%), with a sum of species of 41.8 ± 0.6 μg kg^−1^) and the highest total arsenic content (80.5 ± 0.7 μg kg^−1^). Therefore, Pom-Coll was used for most primary method development experiments. Thirteen additional juices, including cherry (Cherry 1–3), pomegranate (Pomegranate 1–4), prune (Prune 1–4), and blueberry (Blueberry 1–2), were purchased from local markets (College Park, MD) by CFSAN personnel and analyzed to determine if they could be categorized as LMBJ, see Fig. 1 (Pomegranate 4 and Prune 4 were not subjected to speciation analysis). Three of these locally purchased samples were chosen for further analysis and utilized for the MLV study (Prune 2—designated Prun, Pomegranate 3—designated Pom, and Cherry 2—designated Cher). Also, four samples, one apple juice (AJ1), one purple grape juice (GJ1), one white grape juice (GJ3), and one pear juice (PJ3) which were previous analyzed as part of the multilaboratory validation for the EAM 4.10 (Kubachka et al. 2017), were also included in this study.

### Total Arsenic and Arsenic Speciation in Juice

Total arsenic was determined by the CFSAN laboratory using the FDA EAM method §4.7 (EAM 4.7) (Gray et al. 2015). In summary, samples were subjected to microwave-assisted digestion with concentrated nitric acid, diluted, and analyzed with an online internal standard against appropriate external calibration standards using the ICP-MS. Initial speciation work for all juice samples was performed using the EAM 4.10. In summary, samples were diluted with DIW and analyzed with a post-column marker (instrument drift standard) against appropriate external calibration standards using the HPLC-ICPMS. The speciation and total arsenic concentrations were compared to classify samples as LMBJs. For method development, samples were made up in either DIW or various concentrations of dilute nitric acid, with or without heating. As previously mentioned, unless otherwise noted, the EAM 4.10 chromatographic conditions were used for all analyses. For flow injection analysis, the injection of a post-column marker peak was delayed until 2 min to avoid interference from the sample injection peak (sample injected without an analytical column).

### Multilaboratory Validation

After finalizing method development, the optimized sample preparation procedure was subjected to a level 3 validation according to the FDA guidance (Guidelines for the Validation of Chemical Methods for the FDA FVM Program 2015) executed by the KAN and SAN laboratories. To fulfill requirements of a level 3 MLV, three juice matrices were analyzed in triplicate; three fortified analytical portion (FAP) levels for each matrix were analyzed in duplicate. Samples were shipped with cold packs and refrigerated upon receipt until used. The samples were only analyzed for speciation by the validation laboratories, while the total arsenic numbers determined by the CFSAN laboratory were used to evaluate the mass balance. The laboratories were instructed to follow the modified sample preparation procedure while performing analysis procedures as described in the EAM 4.10, including quality control (QC) parameters. The FAPs were prepared at 8, 16, and 32 μg kg^−1^ for each As(III), DMA, MMA, and As(V). The laboratories were instructed to report any FAPs that failed but were not required to reanalyze unless there were multiple QC failures or other concrete reasons for doing so. At the time of the MLV study, there were no standard reference materials for juice; therefore, the SRM 1643f was prepared along with the juice samples, but without FAPs. Additionally, method blanks and fortified method blanks were analyzed as additional quality controls.

## Results and Discussion

### Initial Analysis of Juices

The sample identified as Pom-Coll was determined to have a total arsenic concentration of 80.5 μg kg^−1^. Initial analysis by the EAM 4.10 resulted in 41.8 μg kg^−1^ of iAs with no other species detected for a mass balance of 51.9%. As previously mentioned, this juice was the focus of initial method development steps. To determine if other juices exhibited this trait, additional juices were tested, with the premise that the darker juices may have this problem. However, previous results indicate that purple grape juice is an exception to this trend, as quantitative mass balances are frequently reported (Conklin and Chen 2012; Kubachka et al. 2017; Narukawa et al. 2018). The previously mentioned locally purchased juices were analyzed for both total arsenic and speciation using the EAM 4.10, see Fig. 1. For both analyses, all samples were analyzed within the same analytical sequence with one sample prepared in triplicate and the remaining ten were analyzed in singlet. Mass balances for all prune, pomegranate, and cherry juices ranged from 44 to 80% (59 ± 13%), while both blueberry juice samples had a 91% recovery. Although data was limited, blueberry juice was not categorized as a LMBJ; therefore, it was not analyzed further. Cherry juice samples had the next highest mass balance, but two of the 3 were below 75% and were considered LMBJ. Prune 2 (Prun), Pomegranate 3 (Pom), and Cherry 3 (Cher) were selected for further method development analysis as they represented each matrix with a mass balance issue (LMBJ) and a general total arsenic concentration range of 10–40 μg kg^−1^.

### Method Development to Improve Mass Balance

One potential cause for low mass balances was surmised to be a result of the juice filtration step. Three juice samples, Prune 4, Pomegranate 4, and Cherry 5 (only Cherry 5 was also speciated, see Fig. 1), were unfiltered or filtered then subjected to total arsenic analysis by ICPMS. The filtered portions of these juices yielded total arsenic concentrations of 99%, 89%, and 83%, respectively, compared with the unfiltered juice. Although a portion of the arsenic may be retained during filtering as some of the juices exhibited visible particulate trapped on the filter, the discrepancy was not sufficient to explain the full loss of arsenic as shown in Fig. 1; therefore, additional options were explored; unless otherwise noted, samples were filtered for the subsequent analyses.

Another possible cause of low mass balances was that arsenic was precipitating out of solution during dilution of the sample, prior to analysis. Therefore, the Pom-Coll, Prun, Pom, and Cher samples were diluted (following the EAM 4.10) and injected on the column, with results similar to dashed columns in Fig. 1. The exact analytical solutions analyzed via the EAM 4.10 were injected again, but without an analytical column (flow injection (FI) analysis). Initially, the peak areas were compared, but this can be misleading as other juice components (in particular carbon-containing (Larsen and Sturup 1994)) could enhance the arsenic signal when doing flow injection. Therefore, standard additions of As(V) at approximately 2, 4, and 6 times the total arsenic concentration of the specific sample were prepared and analyzed by the FI-ICPMS. The total arsenic of the sample was delineated using the method of standard additions and compared with the sum of the species using the EAM 4.10. The results are shown in Table 1. The flow injection (FI-ICPMS) total arsenic concentrations were similar (within ± 20%) to the total arsenic analysis for samples previously designated Pom-Coll, Prun, Pom, and Cher, indicating that the arsenic does not precipitate out of solution and is being injected into the HPLC system. The low column recoveries imply that the analytes are not eluting from the analytical column as expected.

The stationary phase of the PRP-X100 is poly(styrene-divinylbenzene) with trimethyl ammonium and exhibits anionic exchange properties as well as some hydrophobic characteristic which may lead to retention of larger hydrophobic compounds. Altering the chromatography was considered a last resort, as keeping the method as similar to the EAM 4.10 was a priority, but several alternative separations were investigated to attempt to understand the low mass balance, as explained later in this manuscript. Perhaps the arsenic being bound to a larger compound/complex could explain the poor column recovery and subsequently low mass balance. However, common effects of column overloading such as increased back pressure or decreased separation efficiency were not noted. Although not always ideal, using a sample preparation that converts the *in situ* arsenic complex to an arsenic species that elutes from the column might provide clues as to the source of the missing arsenic.

**Fig. 2:**
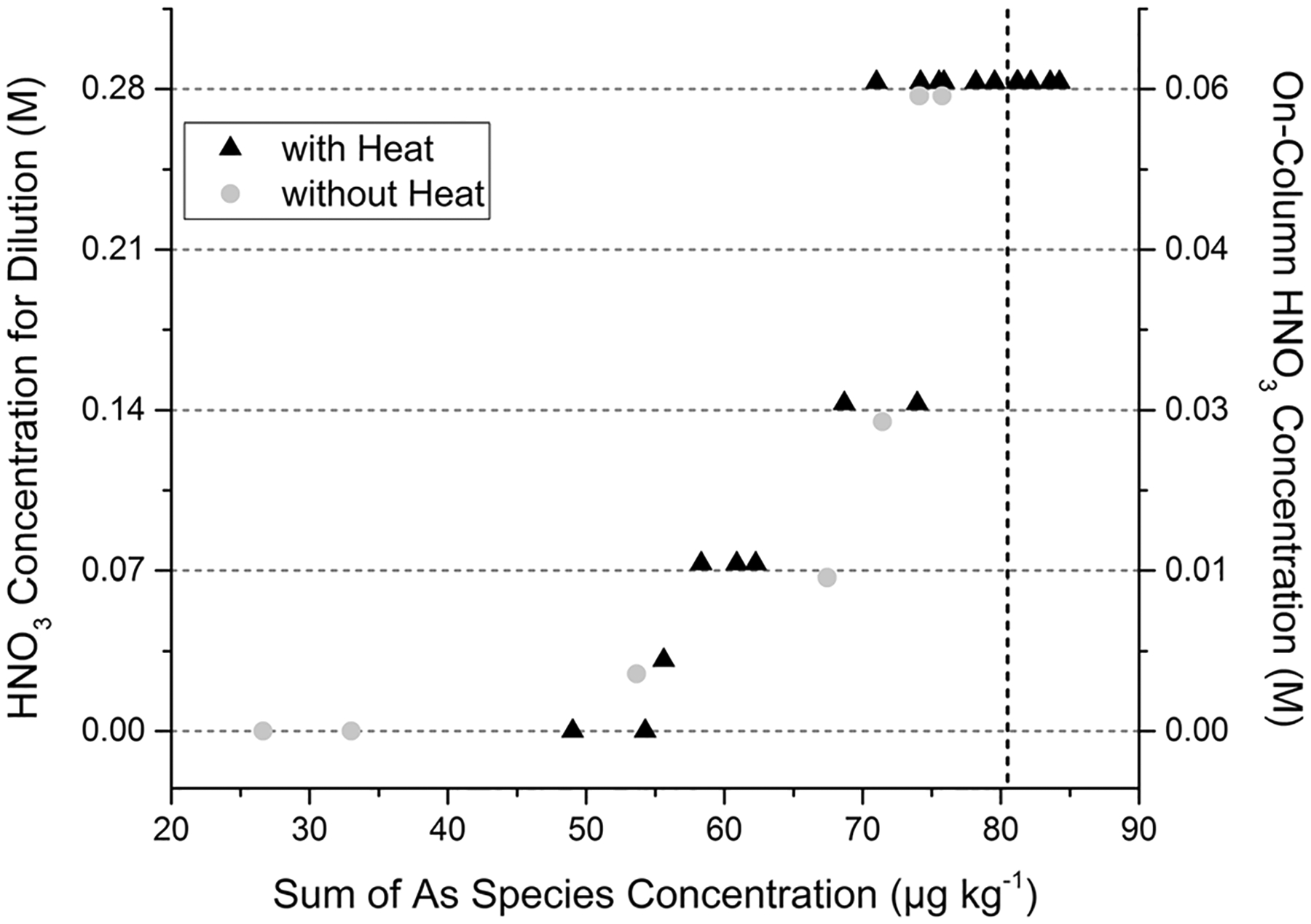
Recovery of iAs (i.e., mass balance as only iAs species were detected) versus dilution with increasing concentrations (and on-column equivalent) of dilute HNO_3_ solutions, without (gray circles) and with heat (95 °C for 90 min, black triangles). Vertical dashed line represents total As concentration of Pom-Coll juice sample previously determined by ICP-MS

As simple dilution does not alleviate this issue, additional sample preparation alterations were explored. As previously mentioned, other reports utilized nitric acid solutions for dilution/digestion of beverages (Ellingson et al. 2014; Roberge et al. 2009) while a common arsenic speciation extraction for food matrices, including rice, utilized dilute nitric acid and heat (Huang et al. 2010). Such an extraction is implemented in the FDA EAM method §4.11 (EAM 4.11) for rice and rice products (Kubachka et al. 2015); therefore, this offers a practical solution because the supplies and general steps for extraction may already be familiar to analysts using similar methodology. Based on these reasons, it was initially attempted to extract the arsenic by simple five-fold dilution (similar to that of the EAM 4.10) of Pom-Coll with dilute nitric acid (0, 0.028, 0.07, 0.14, and 0.28 M solutions), shown as gray circles in Fig. 2. A 2-g aliquot of the juice was mixed with 2 g of the acid solution, then diluted to 10 g with DIW. To normalize solution concentration for subsequent comparisons, the dilutions will be also listed with their “on-column” nitric acid molarity. The initial experiments using the aforementioned dilutions (on-column HNO_3_ concentrations of 0, 0.006, 0.014, 0.028, and 0.056 M, respectively) show that as the nitric acid concentration increases, so does the sum of the arsenic species recovered from the column. Although there is difficulty controlling the As(III)/As(V) interconversion, the arsenic compound that significantly increases with the increasing acid content is As(V). Therefore, it can be inferred that the acid liberated As(V) from Pom-Coll matrix allowing it to elute from the column. The highest average mass balance came from the dilution with 0.28 M HNO_3_ (0.056 M on column HNO_3_ concentration) at 93 ± 1% (*n* = 3). Although this represents a vast improvement versus dilution with water, attempts were made to further increase the mass balance. Increasing the HNO_3_ concentration of the dilution solution beyond 0.28 M (0.056 M on column) could be an option, but too much HNO_3_ was deleterious to both the column (Huang et al. 2010) and the separation. Although the nitric acid concentration used for extraction is significantly less than typically used for total arsenic analysis (15.8 M), it is worth noting that approaching higher concentrations will increase the potential conversion of arsenic species to inorganic arsenic. Therefore, rather than increasing the acid concentration, the addition of heat was explored. A 2-g aliquot of the juice was mixed with 2 g of the acid solution, heated on a hot block at 95 °C for 90 min (to align with existing FDA methodologies), then diluted to 10 g with DIW. The temperature and time were not fully optimized for juice as lower times and temperatures may be sufficient. When doing so, the arsenic recoveries increased further, particularly for 0.28 M (0.056 M on column) dilution as shown in Fig. 2, black triangles. The average mass balance was 98 ± 5% (*n* = 11)

### Effects on Chromatography

**Fig. 3:**
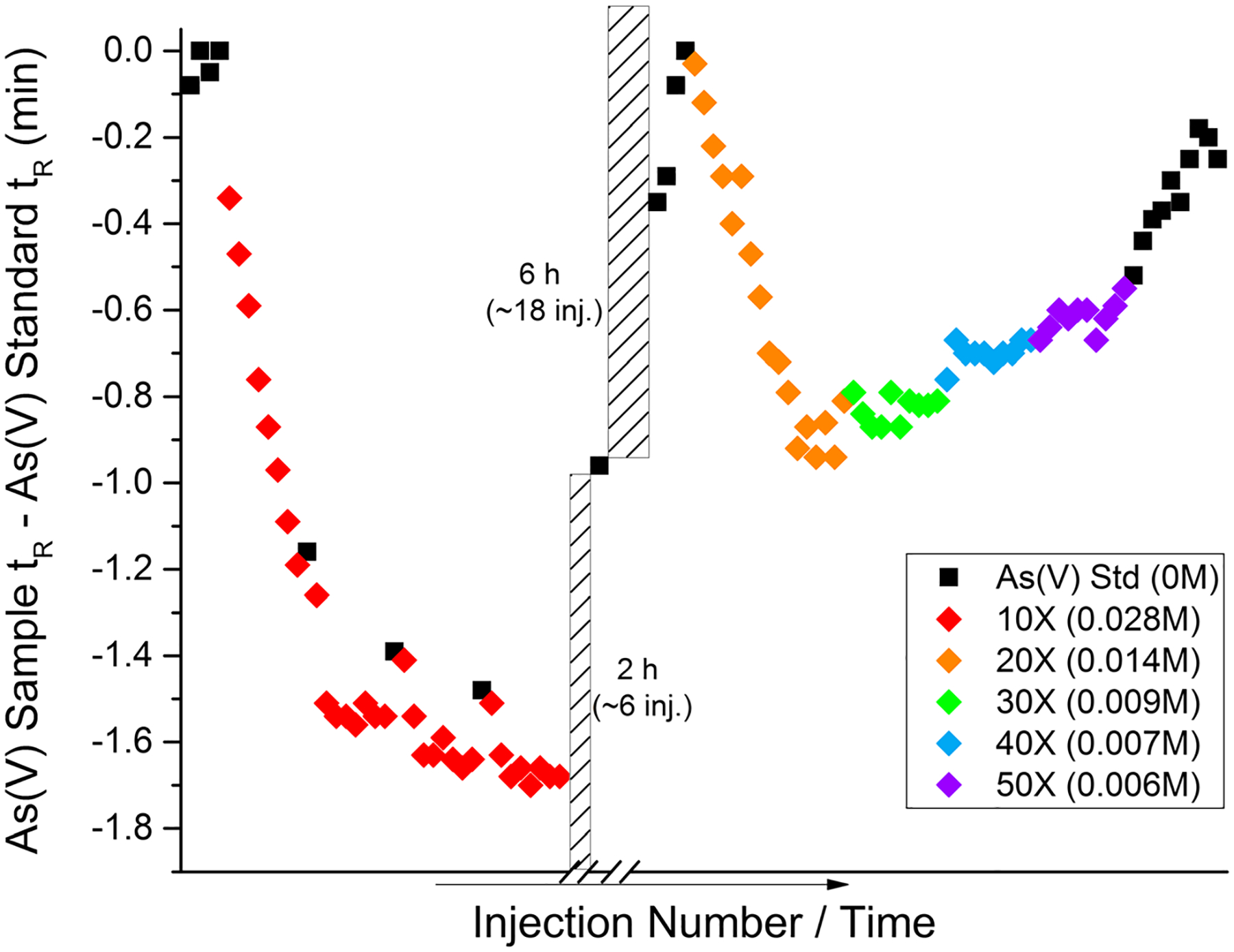
As(V) retention time shift versus injection order as a function of on-column HNO_3_ concentration (M). Injections were continuous except two noted gaps (dashed bars), during which the mobile phase was flowing through the column at 1.0 mL min^−1^ and no injections were made. Each injection was approximately 20 min, and all data points were generated using the same HPLC-ICP-MS setup and conditions running continuously over 2 days

As mentioned, one noted problem was the decrease in As(V) retention time and to a lesser extent MMA. The data displayed in Fig. 2 was based on a five-fold dilution with the various HNO_3_ solutions; however, the As(V) peak eluted at 13.5 min (0.056 M HNO_3_ on-column) compared with an As(V) containing standard in DIWanalyzed earlier in the same analytical sequence in which As(V) eluted at 17.0 min. With a relatively large shift in retention time, this could lead to peak misidentifications. Peak identifications could be addressed with fortified analytical solutions or fortified analytical portions (FAP), but minimization of retention shift would be preferable. To determine the cause of the retention time shift, the pH of the sample (< 2) was adjusted with NH_4_OH to 3.8 and further to 7.7 (near the pH of the mobile phase). The retention time decreased further, therefore indicating that the increase in ionic strength is the likely factor for the shift, rather than the pH of the analytical solution. This could be addressed by diluting the sample further to limit the retention time shift. As previously mentioned, a five-fold dilution was not sufficient; therefore, dilutions of ~ 10- to 50-fold (0.028 to 0.006 M HNO_3_ on column, respectively) were attempted. Due to differences in column performance from column age to usage, retention time shifts (Δ*t*_R_) will be expressed as the analyte *t*_R_ of the initial injection (per analysis day) of an arsenic mix including 2 μg kg^−1^ each of As(III), DMA, MMA, and As(V) in DIW minus the analyte *t*_R_ of subsequent diluted juice extracts. After the initial arsenic standard injection, samples were injected, from lowest to highest dilution repeatedly until the As(V) *t*_R_ became stable; then, the arsenic standard was injected to determine if the column was irreversibly damaged or if the retention time would eventually approach its original time. This injection pattern was repeated with increasing dilutions as shown in Fig. 3. Although all dilutions produced a noticeable shift, there was a drastic reduction in As(V) Δ*t*_R_ from the 10-fold dilution (− 1.7 min) to the 20-fold dilution (− 0.9 min). The change in As(V) Δ*t*_R_ was less significant going from 20-to 50-fold dilutions. The approximate shift for the 20-fold dilution was about − 0.9 min, while the 50-fold resulted in a − 0.6-min shift.

### Dilution Factor and Analytical Limits

The next consideration was to balance dilution factor (and retention time) with detection/quantitation limit. The LOD and LOQ for the EAM 4.10 are 0.25 and 2.0 μg kg^−1^, respectively. Note that LOD and LOQ are based on the FDA EAM 3.2 (Cunningham et al. 2014) which estimates a conservative LOQ of 30*σ* (2.0 μg kg^−1^) versus the typical 10*σ* (LOQ of 0.67 μg kg^−1^). As these are based on a five-fold dilution, the LOD and LOQ for the adjusted procedure would be higher in accordance to the final dilution factor. Currently, there are no regulations for iAs in any of the LMBJ juices (prune, pomegranate, or cherry), but using the proposed action limit (FDA 2013) of 10 μg kg^−1^ iAs in apple juice as a benchmark, it would be ideal to keep the LOQ below 10 μg kg^−1^. Therefore, a 20-fold dilution provides a good balance as the retention time shift (< 1 min for As(V)) is reasonable, and the LOD and LOQ are 1 and 8 μg kg^−1^, respectively (10*σ* LOQ of 2.7 μg kg^−1^).

For the optimized sample preparation (referred herein as extraction) conditions, 5 g of juice was weighed into a 50-mL PTFE centrifuge tube and 6 g of 0.28 M HNO_3_ was added. The tube was tightly capped, and the mixture was heated at 95 °C for 90 min. After cooling, 9 g of DIW was added (total weight of 20 g), followed by centrifugation (optional, to collect visible particulates at the bottom) and filtration with a 0.45-μm syringe filter (Nylon or PTFE). The filtered portion (first mL discarded) was diluted five-fold with DIW and placed into a plastic autosampler vial for analysis by the HPLC-ICPMS. The final dilution factor of the optimized extraction procedure was 20.

### Method Robustness

To examine the robustness of the extraction method, three laboratories analyzed the samples with deviations from the optimized extraction conditions. All laboratories used 5 g of sample. One laboratory (lab 1) used the following different extraction conditions: 8 g of 0.28 M HNO_3_; after heating, 7 g of DIW for the initial dilution; and 1 mL of extract diluted five-fold with DIW for a final dilution factor of 20. The second laboratory (lab 2) used the following different extraction conditions: 10 g of 0.28 M HNO_3_; after heating, 1.6 g of DIW for the initial dilution; and 1 mL of extract diluted threefold with mobile pH with a pH of 9.9 (to increase the pH of the analytical solution) for a final dilution factor of 10. Additionally, Pom-Coll was analyzed by a third laboratory (lab 3) using these modified conditions: 5 g of 0.28 M HNO_3_; after heating, 15 g of DIW for the initial dilution; and 2 g of extract diluted to 6.5 g with DIW for a final dilution factor of ~ 16. The results for these analyses are shown in Table 2. Overall, the deviations for the extraction procedure provided nearly quantitative mass balances for the samples tested and show that the optimized extraction is robust. One thing to note is that MMA was only detected in Pom when the 10-fold dilution was used, as higher dilutions put it below the LOD. Additionally, the corresponding results for each sample are within 20% of the pooled results from the validation exercise presented in Table 3.

**Fig. 4:**
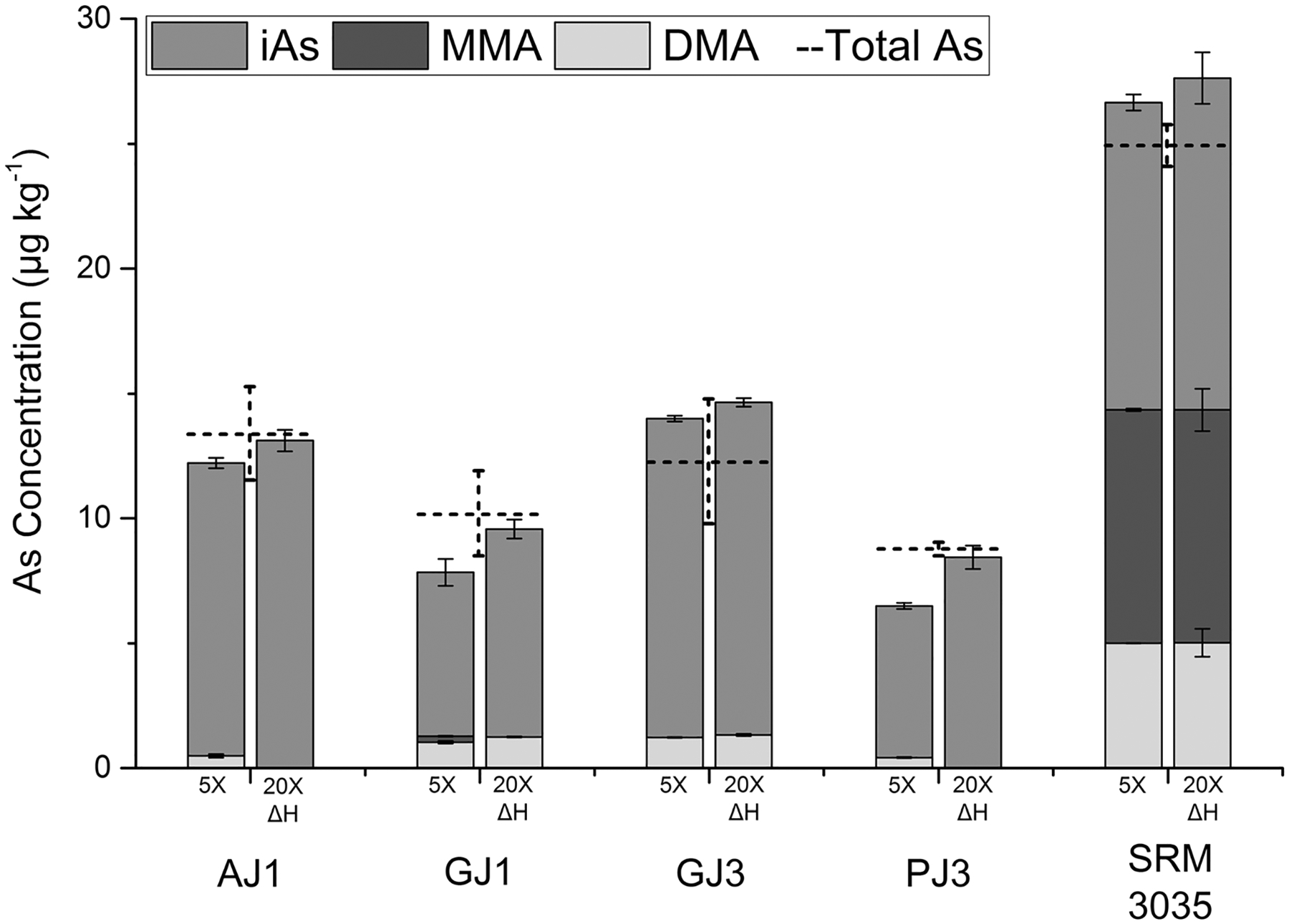
Comparison of arsenic species detected in five juice samples using the original preparation method described in EAM 4.10 (a five-fold dilution, 5×) and the extraction (heated acidic extraction with twenty-fold dilution, 20× ΔH). Concentrations represent the average of 2 or 3 replicates. Dashed bars represent total arsenic concentration. All error bars represent ± *σ*

Four juice samples analyzed as part of the initial MLV for the EAM 4.10, designated AJ1, GJ1, GJ3, and PJ3, along with the NIST SRM 3035 and SRM 1643f, were selected for further analysis. These samples were removed from frozen storage and analyzed again by the EAM 4.10, and results were compared with the extraction. This experiment was done to ensure that juices which do not exhibit significant mass balance deficiencies would produce similar results using both preparation methods. Due to limited amount of these samples, minor adjustments were made to the preparation procedure: sample and reagent amounts were scaled down by a factor of ten, samples were prepared in 15-mL tubes, and heating was performed using an oven. The reanalysis of AJ1, GJ1, GJ3, and PJ3 by the EAM 4.10 compared well with the data from original MLV, therefore demonstrating that storage conditions were effective at preserving the original speciation. As shown in Fig. 4, all the samples, to varying degrees, using the extraction resulted in increased mass balances, primarily due to increased iAs recovery. The two samples with the lowest mass balances of the original EAM 4.10 validation, GJ1 and PJ3 (68 and 66%, respectively), showed the largest improvement to 94 and 96%, respectively. This implies that grape and pear juice types could potentially exhibit low mass balance juice characteristics, but additional samples would need to be analyzed. Analysis of the SRM 3035 (*n* = 3) using the extraction resulted in percentage recoveries compared with the certified values of DMA, MMA, and iAs of 96 ± 11, 98 ± 9, and 115 ± 11%, respectively. When analyzed by using the EAM 4.10 (*n* = 2), the recoveries were 96 ± 0.3, 100 ± 0.5, and 107 ± 3%, for DMA, MMA, and iAs, respectively. All recoveries were within the ± 20% acceptance criteria outlined in the EAM 4.10. The percent recovery of inorganic arsenic detected in the SRM 1643f (*n* = 2) using the extraction was 95 ± 0.2% of the certified total arsenic concentration. Note that for AJ1 and GJ3, DMAwas below the LOD using the 20× dilution and between the LOD and LOQ, in the 5× dilution.

### Column Recovery

As previously mentioned, the sample extraction resulted in an increase in the As(III), and to a greater extent As(V) (noted as an iAs increase), compared with DIW dilution of the same LMBJ. Therefore, it is reasonable to assume that the iAs is natively bound to some type of complex within the juice that elutes from the syringe filter, but does not elute from the PRP-X100 column, and the acidic extract liberates (or converts) the arsenic from this complex so that it elutes as As(III) and/or As(V). The acidic extraction is not harsh enough to break the As–C bonds of DMA and MMA, as evidenced by their successful FAP recoveries. To determine the original form of the missing iAs, additional chromatographic conditions/columns were explored. A separation using only the PRP-X100 guard column (shorter version with same packing material) did not show any additional peaks, even as the injection was monitored for more than 30 min, suggesting a strong interaction with the stationary phase. Even when injecting a sample and using the column regeneration solution as the mobile phase (99% MeOH, 1% 6 M HNO_3_), which should elute all retained species from the column (with the ICP-MS set in organic plasma mode), no additional peaks were observed. Another possibility would be the presence of sulfur analogs of arsenite or arsenate which do not elute readily from the PRP-X100. Therefore, separation conditions similar to what was used by Wallschlager and Stadey (2007) were attempted; however, no increase in analyte recovery was noted. The final set of conditions was to use a size-exclusion column (BioSep SEC-S3000, Phenomenex Torrance, CA). The mass balance and column recovery approached 100%, but the poor peak resolution made additional peaks undiscernible; therefore, additional experiments beyond the scope of this manuscript are necessary to determine the origin of the bound iAs. Based on the fact that this extraction converts an unknown form of arsenic to iAs, it is plausible that other arsenic compounds could be converted. Based on FAP recoveries, data from Huang et al. (Huang et al. 2010), and additional unreported FDA data, the extraction does not show any detectable conversion of DMA and/or MMA to inorganic arsenic.

### Method Validation

The optimized extraction procedure was subjected to a level 3 MLV study (FDA 2015). The three juice matrices chosen for the validation were the previously mentioned samples Prun, Pom, and Cher. Both participating laboratories were familiar with the FDA EAM 4.10 and 4.11 and therefore had all the necessary supplies and reagents. The results for the replicate analyses are shown in Table 3 and the summary of the FAPs are presented in Table 4.

The results show that the iAs arsenic quantitation is reproducible as the highest percentage RSD was < 10%. DMA was only detected at trace levels in the Pom sample but exhibited percentage RSD < 6%. MMA was not detected in any of the samples. The sum of the species compared well with the previously determined total arsenic concentration as mass balances averaged between 86 and 102%. The average mass balance between both laboratories for Cher, Pom, and Prun were 102 ± 4, 93 ± 6, and 93 ± 8%, respectively. The within laboratory relative standard deviations (RSD) for DMA in Pom for labs 3 and 4 were both 5%. The within laboratory RSDs for iAs for all three juices ranged from 0.4 to 9% and 3 to 4%, for labs 3 and 4, respectively. The sum of the species for the SRM 1643f compared with the certified total arsenic values yielded recoveries of 94 ± 2% and 103 ± 1% for the two laboratories.

Between the two laboratories, a total of 36 juice sample FAPs were analyzed (three levels, duplicate preparations, three matrices, for two laboratories) along with 10 fortifications of method blanks, for each analyte. Individual As(III) and As(V) FAP recoveries were calculated as one laboratory reported high As(III) recoveries (> 120%), but low As(V) recoveries (< 80%); this could be explained by antioxidant components in the juice. As noted in Table 4, the iAs recoveries were quite accurate as the average recoveries ranged from 96 to 99% for all three juice types. Only one FAP was outside of the defined QC parameters for the EAM 4.10 (80–120%)—the 8-μg kg^−1^ MMA FAP for the Prun sample, at 139%. Although this may be a statistical outlier, it was not excluded from this data set. Examination of the FAP data shows no significant interspecies conversion was detected, other than between As(III) and As(V).

Method blanks did not include any detectable levels of arsenic, and FMBs (prepared at 16 μg kg^−1^ level) did not have recoveries outside of the 80–120% range. Additionally, both laboratories analyzed the SRM 1643f as a check standard (dilution in DIW, no extraction) and achieved an average recovery of 95% (*n* = 7) versus the certified values. Check standards (*n* = 16) of 2 μg kg^−1^ each of As(III), DMA, MMA, and As(V) had recoveries within 85–115% of the expected concentration.

At the time of the manuscript preparation, the extraction had been applied to at least three additional samples encountered during FDA survey work. All were pomegranate juices with total arsenic concentrations of 36.5 ± 3.5, 11.7 ± 0.2, and 18.1 ± 0.1 μg kg^−1^ and exhibited mass balances implementing the modified extraction procedure (*n* = 3 for each sample) of 89 ± 3, 89 ± 1, and 93 ± 1%, respectively.

## Conclusions

Various juice types referred to herein as low mass balance juices, when diluted with DIW and analyzed by the HPLC-ICPMS (using the EAM 4.10), exhibited a low mass balance (~ 50%). It was determined that the missing arsenic was not eluting from the analytical column, resulting in low column recovery. This was surmised to be due to As(III) and/or As(V) being associated with larger complex that bound to the stationary phase. A modified extraction procedure using 0.28 M HNO_3_ along with heat allowed for the quantitative recovery of the arsenic via speciation analysis. The presented preparation, which appears to liberate bound iAs from the juice matrix, provides a more conservative approach from a risk assessment view, as all potential iAs is detected, but may not be bioavailable.

The extraction leads to nearly quantitative recovery of the arsenic species of interest for samples identified as LMBJ. Upon optimizing the extraction parameters, two laboratories performed a level 3 MLV exercise for cherry, pomegranate, and prune juice yielding average mass balances of 102, 93, and 93%, respectively. Additionally, analysis of the only available juice SRM for arsenic species and the SRM 1643f yielded acceptable results, further supporting the accuracy of this preparation procedure. Regarding the reproducibility of the method, relative standard deviations were less than 10% for iAs for all samples analyzed. For DMA, RSDs were similar (< 15%) even though the majority of DMA concentrations were near or below the LOQ. Due to the inherently higher LOD and LOQ from the increased dilution factor (5-fold versus 20-fold), this preparation method is not aimed to replace that described in the EAM 4.10, but to be used in tandem. Ideally, this modified preparation should be implemented when total arsenic values of a juice are above 10 μg kg^−1^, but mass balances (based on the EAM 4.10 analysis) are < 75%. It is planned that this alternate sample preparation procedure will be added as an appendix to the EAM 4.10.

## Figures and Tables

**Table 1:** Comparison of total arsenic by ICP-MS, total arsenic by FI-ICPMS, and sum to species by HPLC-ICPMS (EAM 4.10) as it relates to extraction efficiency and column recovery. All values are averages ± 1*σ*, *n* = 3 unless otherwise noted

Sample	ICP-MS total As (μg kg^−1^)	Flow injection (FI-ICPMS) total As (μg kg^−1^)	FI-ICPMS/total As (%)	EAM 4.10 sum of species (μg kg^−1^)^a,^	Column recovery, EAM 4.10 sum of species/FI-ICPMS (%)
Prune 2 (Prun)	31.6 ± 0.1^a^	36.7 ± 4.9	116	16.7 ± 0.2^b^	46
Pomegranate 3 (Pom)	22.7 ± 0.1^a^	23.6 ± 1.2	104	11.7 ± 0.2^b^	50
Cherry 3 (Cher)	10.6 ± 0.1^a^	10.0 ± 0.31	94	7.72 ± 0.2^b^	76
Pom-Coll	80.5 ± 0.7^c^	76.7 ± 2.7	95	41.8 ± 0.6	55

a Pom analyzed in triplicate; standard deviation was also applied to all others analyzed in same batch, *n* = 1

b Prun analyzed in triplicate; standard deviation was also applied to all others analyzed in same batch, *n* = 1

c *n* = 2

**Table 2:** Speciation results obtained by three laboratories, each implementing deviations from the optimized extraction procedure to evaluate the robustness of the sample extraction procedure. Total As determined by ICP-MS; DMA, MMA, and iAs were determined by HPLC-ICP-MS

Sample	Total As^a^ (μg kg^−1^)	Lab (replicates)	DMA (μg kg^−1^)	MMA (μg kg^−1^)	iAs (μg kg^−1^)	Mass balance (%)
Prune 2 (Prun)	31.6 ± 0.1	1 (*n* = 2)	< LOD	< LOD	29.9 ± 3.0	95
		2 (*n* = 3)^b^	0.3 ±0.1	< LOD	27.7 ± 0.7	89
Pomegranate 3 (Pom)	22.7 ± 0.1	1 (*n* = 2)	4.4 ±0.5	< LOD	20.7 ± 0.1	111
		2 (*n* = 1)^b^	3.0 ±0.1	0.3	15.8 ± 0.7	84
Cherry 3 (Cher)	10.6 ± 0.1	1 (*n* = 2)	< LOD	< LOD	12.7 ± 1.4	120
		2 (*n* = 1)^b^	0.2 ±0.1	< LOD	10.1 ± 0.7	98
Pom-Coll	80.5 ± 0.7	3 (*n* = 3)	< LOD	< LOD	80.9 ± 3.8	101

iAs, μg kg^−1^ = As(III), μg kg^−1^ + As(V), μg kg^−1^

a Values from Table 1

b Prun analyzed in triplicate; standard deviation was also applied to all others analyzed in same batch, *n* = 1

**Table 3:** Arsenic speciation results of MLV for each of the three samples prepared using the optimized extraction procedure and analyzed by HPLC-ICPMS following procedures in EAM 4.10

Lab	Sample	iAs (μg kg^−1^)^a^	DMA (μg kg^−1^)^a^	Sum of As species (μg kg^−1^)^a^	Mass balance^b^
3	Prun	27.1 ± 0.1 (0.4% RSD)	< LOD	27.1 ± 0.14 (0.4% RSD)	86 ± 0.3%
	Pom	18.6 ± 1.7 (9% RSD)	2.30 ± 0.11 (5% RSD)	20.9 ± 1.8 (9% RSD)	92 ± 8%
	Cher	10.8 ± 0.6 (5% RSD)	< LOD	10.8 ± 0.6 (5% RSD)	102 ± 5%
4	Prun	31.6 ± 1.4 (4% RSD)	< LOD	31.6 ± 1.4 (4% RSD)	100 ± 4%
	Pom	18.8 ± 0.8 (4% RSD)	2.40 ± 0.12 (5% RSD)	21.2 ± 0.9 (4% RSD)	94 ± 4%
	Cher	10.8 ± 0.3 (3% RSD)	< LOD	10.8 ± 0.3 (3% RSD)	102 ± 3%
Overall	Prun	29.3 ± 2.6 (9% RSD)	< LOD	29.3 ± 2.6 (9% RSD)	93 ± 9%
	Pom	18.7 ± 1.9 (6% RSD)	2.35 ± 0.12 (5% RSD)	21.1 ± 1.3 (6% RSD)	93 ± 6%
	Cher	10.8 ± 0.4 (4% RSD)	< LOD	10.8 ± 0.4 (4% RSD)	102 ± 4%

iAs, μg kg^−1^ = As(III), μg kg^−1^ + As(V), μg kg^−1^

Sum of As species = iAs + DMA, no MMA was detected > LOD

a Mean ± 1*σ* for each laboratory *n* = 3, overall *n* = 6

b Mass balance = sum of As species/total arsenic

**Table 4:** Fortified analytical portions recovery summary for all samples analyzed as part of method validation

Sample	FAP % recoveries			Nominal FAP levels^a^
	iAs $\bar{x}$ (min-max)	DMA $\bar{x}$ (min-max)	MMA $\bar{x}$ (min-max)	
Prun (*n* = 12)	99% (85–112%)	109% (95–116%)	106% (89–139%)	8, 16, 32 μg kg^−1^
Pom (*n* = 12)	96% (80–103%)	103% (99–108%)	97% (88–107%)	8, 16, 32 μg kg^−1^
Cher (*n* = 12)	98% (86–118%)	108% (99–119%)	99% (90–116%)	8, 16,32 μg kg^−1^
Method blank (*n* = 10)	102% (89–112%)	104% (92–119%)	102% (92–112%)	16 μg kg^−1^

iAs, μg kg^−1^ = As(III), μg kg^−1^ + As(V), μg kg^−1a^ As(III) and As(V) were each spiked into the sample at the level indicated
